# Supplementary material for: Metabolic Profiling by GC-MS, In Vitro Biological Potential, and In Silico Molecular Docking Studies of Verbena officinalis
Source: Molecules. 2022 Oct 8;27(19):6685. doi: 10.3390/molecules27196685 (PMC9573548; doi:10.3390/molecules27196685)
Supplement: Supplementary file 1 [file molecules-27-06685-s001.zip › molecules-1914015-supplementary.pdf]

molecules

MDPI

Article

Metabolic Profiling by GC-MS, *In Vitro* Biological Potential, and *In Silico* Molecular Docking Studies of *Verbena officinalis*

Rabia Nisar [1,2], Saeed Ahmad [1], Kashif-ur-Rehman Khan [1,*], Asmaa E. Sherif [3,4], Fawaz Alasmari [5], Afaf F. Almuqati [6], Chitchamai Ovatlarnporn [7], Mohsin Abbas Khan [1], Muhammad Umair [8,*], Huma Rao [1], Bilal Ahmad Ghalloo [1], Umair Khurshid [1], Rizwana Dilshad [1], Khaled S. Nassar [9] and Sameh A. Korma [10,11]

1 Department of Pharmaceutical Chemistry, Faculty of Pharmacy, The Islamia University of Bahawalpur, Bahawalpur 63100, Pakistan
2 Department of Chemistry and Physics, College of Sciences and Mathematics, Arkansas State University, Jonesboro, AR 72404, USA
3 Department of Pharmacognosy, College of Pharmacy, Prince Sattam bin Abdul Aziz University, Alkharj 11942, Saudi Arabia
4 Department of Pharmacognosy, Faculty of Pharmacy, Mansoura University, Mansoura 35516, Egypt
5 Department of Pharmacology and Toxicology, College of Pharmacy, King Saud University, Riyadh 11451, Saudi Arabia
6 Department of Pharmaceutical Chemistry, College of Pharmacy, University of Hafr Al-Batin, Hafr Al-Batin 31991, Saudi Arabia
7 Department of Pharmaceutical Chemistry, Faculty of Pharmaceutical Sciences, Prince of Songkla University, Hat Yai 90110, Thailand
8 Department of Food Science and Engineering, College of Chemistry and Engineering, Shenzhen University, Shenzhen 518060, China
9 Department of Food, Dairy Science and Technology, Faculty of Agriculture, Damanhour University, Damanhour 22516, Egypt
10 Department of Food Science, Faculty of Agriculture, Zagazig University, Zagazig 44519, Egypt
11 School of Food Science and Engineering, South China University of Technology, 510641 Guangzhou, China
* Correspondence: kashifur.rahman@iub.edu.pk (K.-u.-R.K.); umair_uaf@hotmail.com (M.U.); Tel.: +92-3366708638 (K.-u.-R.K.); +86-17712915202 (M.U.)

Table S1. GC-MS study of a methanolic extract (CRVO), *n*-hexane extract (NHVO), ethyl acetate extract (EAVO) and *n*-butanol extract (NBVO) of *Verbena officinalis*.

No.	RT (min)	Peak Area (%)	Name of Compound	Molecular Formula	Molecular Weight (g/mol)
**The compounds identified in methanolic extract (CRVO) of *V. officinalis***					
1	6.125	0.43	Benzyl alcohol	C_7H_8O	108
2	6.839	1.07	Benzoic acid, methyl ester	$C_8H_8O_2$	136
3	7.526	0.47	3-Octyne, 7-methyl-	C_9H_{16}	124
4	8.058	0.54	1,4:3,6-Dianhydro-α-d-glucopyranose	$C_6H_8O_4$	144
5	8.685	0.36	3,5-Heptadienal, 2-ethylidene-6-methyl-	$C_{10}H_{14}O$	150
6	8.834	0.36	Phenol, 2-methyl-5-(1-methylethyl)-	$C_{10}H_{14}O$	150
7	8.920	0.46	Indole	C_8H_7N	117
8	10.881	0.59	Undecanoic acid, 10-methyl-, methyl ester	$C_{13}H_{26}O_2$	214
9	11.111	0.64	2(4H)-Benzofuranone, 5,6,7,7a-tetrahydro-4,4,7a-trimethyl-, (R)-	$C_{11}H_{16}O_2$	180
10	11.825	0.24	Megastigmatrienone	$C_{13}H_{18}O$	190
11	12.483	2.57	Methyl tetradecanoate	$C_{15}H_{30}O_2$	242

12	12.576	0.34	2,6-Diisopropylnaphthalene	$C_{16}H_{20}$	212
13	12.643	0.26	1,4-Di-iso-propylnaphthalene	$C_{16}H_{20}$	212
14	12.795	0.22	Caparratriene	$C_{15}H_{26}$	206
15	13.056	0.57	Methyl 13-methyl tetradecanoate	$C_{16}H_{32}O_2$	256
16	13.180	1.59	6-Octadecenoic acid, methyl ester, (Z)-	$C_{19}H_{36}O_2$	296
17	13.275	0.59	9-Octadecenoic acid (Z)-, methyl ester	$C_{19}H_{36}O_2$	296
18	13.360	0.26	11,13-Dimethyl-12-tetradecen-1-ol acetate	$C_{18}H_{34}O_2$	282
19	13.590	0.40	Neophytadiene	$C_{20}H_{38}$	278
20	13.669	0.90	2-Pentadecanone, 6,10,14-trimethyl	$C_{18}H_{36}O$	268
21	13.869	0.26	8,10-Dodecadien-1-ol, (E,E)-	$C_{12}H_{22}O$	182
22	14.164	0.36	Pentadecanoic acid, 14-methyl-, methyl ester	$C_{17}H_{34}O_2$	270
23	14.391	0.54	Methyl hexadec-9-enoate	$C_{17}H_{32}O_2$	268
24	14.653	39.93	Hexadecanoic acid, methyl ester	$C_{17}H_{34}O_2$	270
**The compounds identified in *n*-hexane extract (NHVO) of *V. officinalis***					
**No.**	**RT (min)**	**Peak Area (%)**	**Name of Compound**	**Molecular Formula**	**Molecular Weight (g/mol)**
1	8.017	0.71	Benzoic acid, methyl ester	$C_8H_8O_2$	136
2	8.447	0.30	Octanoic acid, methyl ester	$C_9H_{18}O_2$	158
3	9.466	0.09	Cyclododecane	$C_{12}H_{24}$	168
4	9.505	0.09	2-Decanone	$C_{10}H_{20}O$	156
5	9.962	0.06	Nonanoic acid, methyl ester	$C_{10}H_{20}O_2$	172
6	10.294	0.13	Hexanedioic acid, dimethyl ester	$C_8H_{14}O_4$	174
7	10.941	0.52	Thymol	$C_{10}H_{14}O$	150
8	13.192	0.10	Dimethyl phthalate	$C_{10}H_{10}O_4$	194
9	13.291	0.20	n-hexadecyl methyl imine	$C_{17}H_{35}N$	253
10	13.778	0.08	1,4-Benzenedicarboxylic acid, dimethyl ester	$C_{10}H_{10}O_4$	194
11	13.844	1.90	2,4-Di-tert-butylphenol	$C_{14}H_{22}O$	206
12	13.975	0.56	Dodecanoic acid, methyl ester	$C_{13}H_{26}O_2$	214
13	14.218	0.10	2(4H)-Benzofuranone, 5,6,7,7a-tetrahydro-4,4,7a-trimethyl-, (R)-	$C_{11}H_{16}O_2$	180
14	14.788	0.25	2-Tetradecene, (E)-	$C_{14}H_{28}$	196
15	14.872	0.39	Hexadecane	$C_{16}H_{34}$	226
16	15.166	0.14	Heptadecanoic acid, methyl ester	$C_{18}H_{36}O_2$	284
17	15.329	0.24	Benzene, (1-butylheptyl)-	$C_{17}H_{28}$	232
18	15.632	0.09	1-Nonadecene	$C_{19}H_{38}$	266
19	15.724	1.31	aR-Turmerone	$C_{15}H_{20}O$	216
20	16.019	0.17	Heptadecane	$C_{17}H_{36}$	240
21	16.108	0.22	Benzene, (1-methyldecyl)-	$C_{17}H_{28}$	232
22	16.150	0.28	Curlone	$C_{15}H_{22}$	218
23	16.308	0.56	Methyl tetradecanoate	$C_{15}H_{30}O_2$	242

24	16.388	0.32	Benzene, (1-pentylheptyl)-	$C_{18}H_{30}$	246
25	16.443	0.33	Benzene, (1-butyloctyl)-	$C_{18}H_{30}$	246
26	16.579	0.28	Benzene, (1-propylnonyl)-	$C_{18}H_{30}$	246
27	16.717	0.11	Cyclotetradecane	$C_{14}H_{28}$	196
28	16.817	0.21	Benzene, (1-ethyldecyl)-	$C_{18}H_{30}$	246
29	17.037	0.85	1-Octadecene	$C_{18}H_{36}$	252
30	17.160	3.26	9,12-Octadecadienoic acid (Z,Z)-, methyl ester	$C_{19}H_{34}O_2$	294
31	17.318	12.07	7,10,13-Hexadecatrienoic acid, methyl ester	$C_{17}H_{28}O_2$	264
32	17.442	1.47	6-Octadecenoic acid, methyl ester, (Z)-	$C_{19}H_{36}O_2$	296
33	17.532	1.75	Neophytadiene	$C_{20}H_{38}$	278
34	17.602	1.92	2-Pentadecanone, 6,10,14-trimethyl	$C_{20}H_{38}$	278
35	17.791	0.51	Phytol	$C_{20}H_{40}O$	296
36	17.896	2.36	Heptadecanoic acid, 16-methyl-, methyl ester	$C_{19}H_{38}O_2$	298
37	18.415	12.11	Hexadecanoic acid, methyl ester	$C_{17}H_{34}O_2$	270
38	18.647	0.48	Benzenepropanoic acid, 3,5-bis(1,1-dimethylethyl)-4-hydroxy-, methyl ester	$C_{18}H_{28}O_3$	292
39	18.864	0.42	1-Chloroeicosane	$C_{20}H_{41}Cl$	316
40	19.077	1.19	5-Eicosene, (E)-	$C_{20}H_{40}$	280
41	19.133	0.69	Eicosane	$C_{20}H_{42}$	282
42	19.275	0.28	Henicos-1-ene	$C_{21}H_{42}$	294
43	19.399	0.41	Methyl 10-methyl-hexadecanoate	$C_{18}H_{36}O_2$	284
44	19.528	0.36	Cyclohexane, 1-(1,5-dimethylhexyl)-4-(4-methylpentyl)-	$C_{20}H_{40}$	280
45	19.975	0.50	Nonacos-1-ene	$C_{29}H_{58}$	406
46	20.064	4.92	11,14-Octadecadienoic acid, methyl ester	$C_{19}H_{34}O_2$	294
47	20.124	17.87	9,12,15-Octadecatrienoic acid, methyl ester, (Z,Z,Z)-	$C_{19}H_{32}O_2$	292
48	20.342	2.14	Methyl stearate	$C_{19}H_{38}O_2$	298
49	20.514	0.86	Pentadecafluorooctanoic acid, octadecyl ester	$C_{26}H_{37}F_{15}O_2$	666
50	20.676	0.45	1-Docosene	$C_{22}H_{44}$	308
51	20.939	1.63	Octacosyl acetate	$C_{30}H_{60}O_2$	452
52	21.060	0.54	Pentadec-7-ene, 7-bromomethyl-	$C_{16}H_{31}Br$	302
53	21.485	0.41	3-Eicosene, (E)-	$C_{20}H_{40}$	280
54	21.903	0.76	1-Hexacosene	$C_{26}H_{52}$	364
55	22.033	0.56	9-Tricosene, (Z)-	$C_{23}H_{46}$	322
56	22.701	0.94	Triacontyl acetate	$C_{32}H_{64}O_2$	480
**The compounds identified in ethyl acetate extract (EAVO) of *V. officinalis***					
**No.**	**RT (min)**	**Peak Area (%)**	**Name of Compound**	**Molecular Formula**	**Molecular Weight (g/mol)**
1	5.372	3.07	2-Cyclopenten-1-one, 3-methyl-	C_6H_8O	96
2	6.027	4.44	1-Hexanol, 2-ethyl-	$C_8H_{18}O$	130

3	6.119	0.76	Benzyl alcohol	C_7H_8O	108
4	7.048	0.65	Levoglucosenone	$C_6H_6O_3$	126
5	7.112	1.29	Octanoic acid, methyl ester	$C_9H_{18}O_2$	158
6	7.835	0.79	Benzene, 1-methyl-3-(1-methylethyl)-	$C_{10}H_{14}$	134
7	8.681	1.37	Bicyclo[3.1.1]hept-3-en-2-one, 4,6,6-trimethyl-, (1S)-	$C_{10}H_{14}O$	150
8	8.919	0.85	Benzyl nitrile	C_8H_7N	117
9	9.120	1.06	Decanoic acid, methyl ester	$C_{11}H_{22}O_2$	186
10	10.365	1.60	Dimethyl phthalate	$C_{10}H_{10}O_4$	194
11	10.455	1.09	Cyclododecane	$C_{12}H_{24}$	168
12	10.620	1.34	4-(2,6,6-Trimethylcyclohexa-1,3-dienyl)but-3-en-2-one	$C_{13}H_{18}O$	190
13	10.766	0.47	2-(2-Propenyl)-m-anisidine	$C_{10}H_{13}NO$	163
14	10.878	0.95	Dodecanoic acid, methyl ester	$C_{13}H_{26}O_2$	214
15	11.300	0.61	3-Pyrazolidinone, 4,4-dimethyl-1-phenyl-	$C_{11}H_{14}N_2O_3$	222
16	11.436	0.93	Megastigmatrienone	$C_{13}H_{18}O$	190
17	11.610	0.44	Naphthalene, decahydro-4a-methyl-1-methylene-7-(1-methylethylidene)-, (4aR-trans)-	$C_{15}H_{24}$	204
18	11.821	0.49	1-N-Methyl-4-(trifluoromethyl)benzene-1,2-diamine	$C_8H_9F_3N_2$	190
19	12.315	0.85	2,4,4-Trimethyl-3-(3-oxobutyl)cyclohex-2-enone	$C_{13}H_{20}O_2$	208
20	12.478	2.21	Methyl tetradecanoate	$C_{15}H_{30}O_2$	242
21	13.047	3.96	9,12-Octadecadienoic acid (Z,Z)-, methyl ester	$C_{19}H_{34}O_2$	294
22	13.202	15.38	9,12,15-Octadecatrienoic acid, methyl ester, (Z,Z,Z)-	$C_{19}H_{32}O_2$	292
23	13.665	0.83	2-Pentadecanone, 6,10,14-trimethyl	$C_{18}H_{36}O$	268
24	13.899	0.84	Heptadecanoic acid, 14-methyl-, methyl ester	$C_{19}H_{38}O_2$	298
25	14.647	4.12	Hexadecanoic acid, methyl ester	$C_{17}H_{34}O_2$	270
**The compounds identified in *n*-butanol extract (NBVO) of *V. officinalis***					
**No.**	**RT (min)**	**Peak Area (%)**	**Name of Compound**	**Molecular Formula**	**Molecular Weight (g/mol)**
1	6.028	3.85	2-Cyclopenten-1-one, 3-methyl-	C_6H_8O	96
2	6.108	1.28	3-Heptyne	C_7H_{12}	96
3	6.146	1.75	2,4-Dimethylfuran	C_6H_8O	96
4	7.187	3.56	Phenol, 3-methyl-	C_7H_8O	108
5	8.473	5.95	Octanoic acid, methyl ester	$C_9H_{18}O_2$	158
6	10.778	0.69	Hexadecane, 2,6,10,14-tetramethyl-	$C_{20}H_{42}$	282
7	13.863	1.27	2,4-Di-tert-butylphenol	$C_{14}H_{22}O$	206
8	18.158	0.58	Eicosane	$C_{20}H_{42}$	282
9	18.434	2.90	Hexadecanoic acid, methyl ester	$C_{17}H_{34}O_2$	270

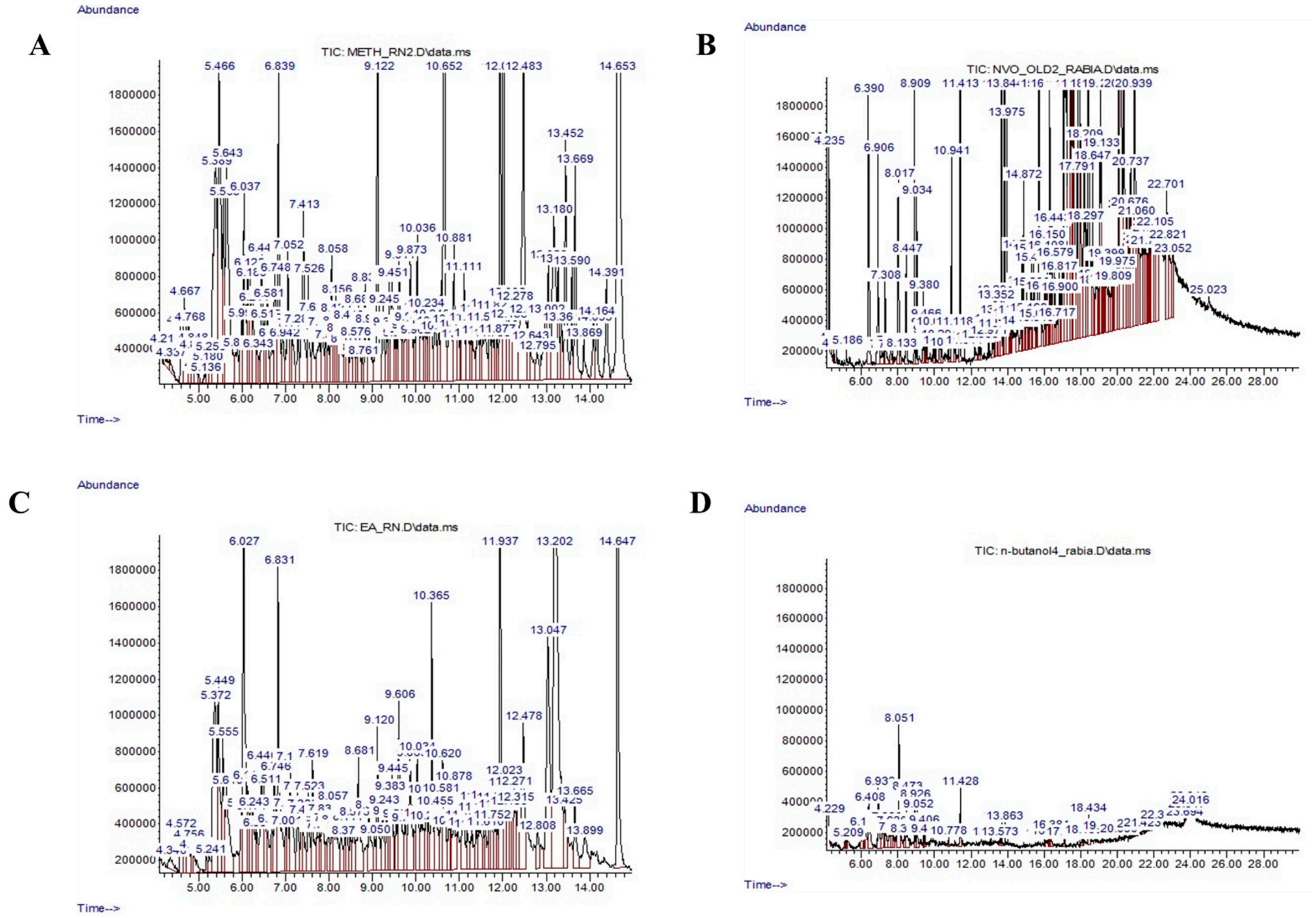

Figure S1. GC-MS chromatogram of (**A**) methanolic crude extract (CRVO), (**B**) *n*-hexane extract (NHVO), (**C**) ethyl acetate extract (EAVO) and (**D**) *n*-butanol extract (NBVO) of *Verbena officinalis*.
